# Supplementary material for: Soft micromachines with programmable motility and morphology
Source: Nat Commun. 2016 Jul 22;7:12263. doi: 10.1038/ncomms12263 (PMC5512624; doi:10.1038/ncomms12263)
Supplement: Supplementary Information — Supplementary Figures 1-10, Supplementary Tables 1-2, Supplementary Notes 1-3 and Supplementary References [file ncomms12263-s1.pdf]

Supplementary Figures and Tables

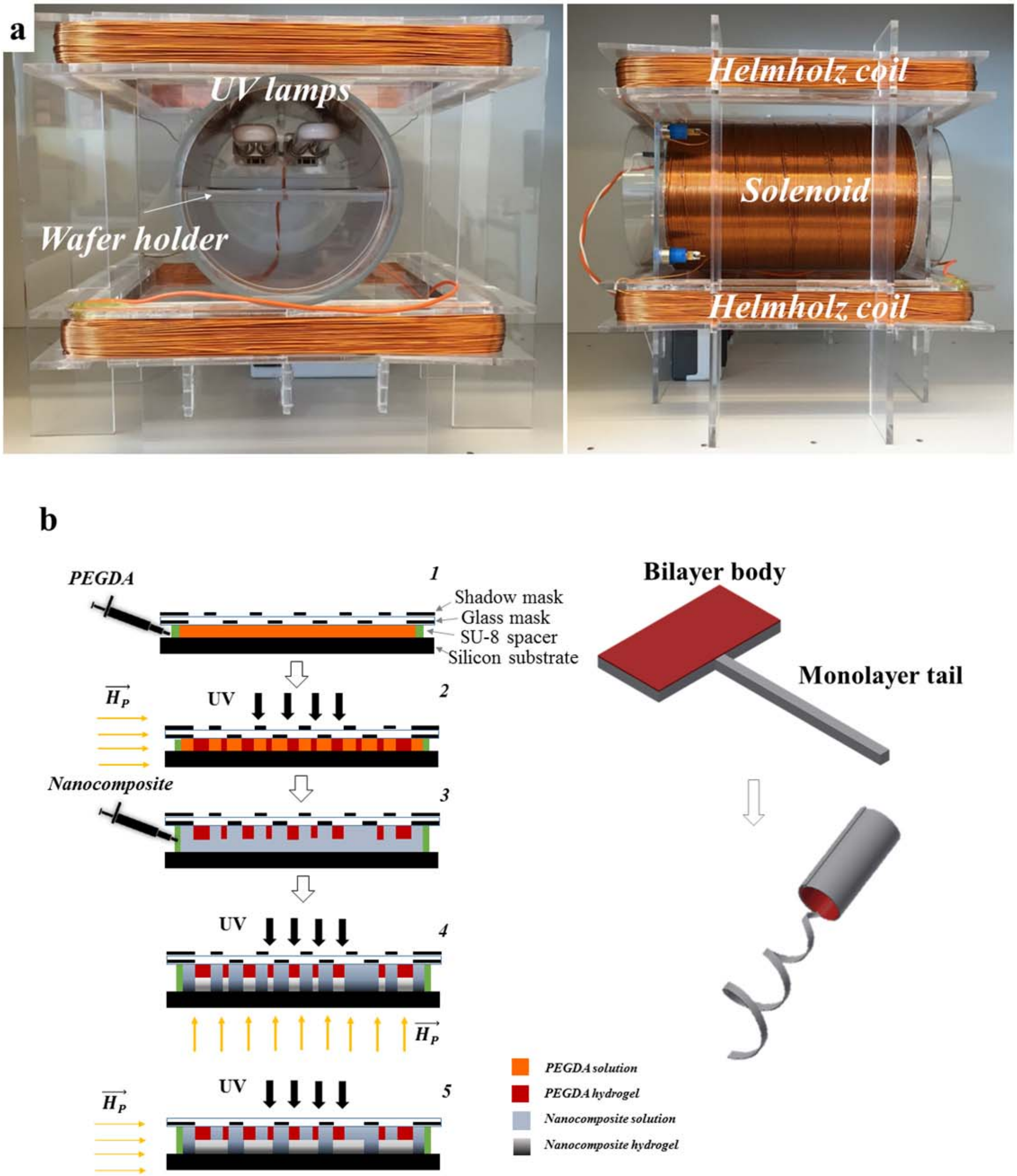

Supplementary Figure 1. Experimental setup and microfabrication processes. (**a**) Images of the experimental setup. While the electromagnetic coils generate uniform magnetic fields for the alignment of magnetic nanoparticles, UV lamp initiates the photo-polymerization of hydrogel nanocomposites. (**b**) Schematic description of the fabrication processes for building hybrid soft micromachines.

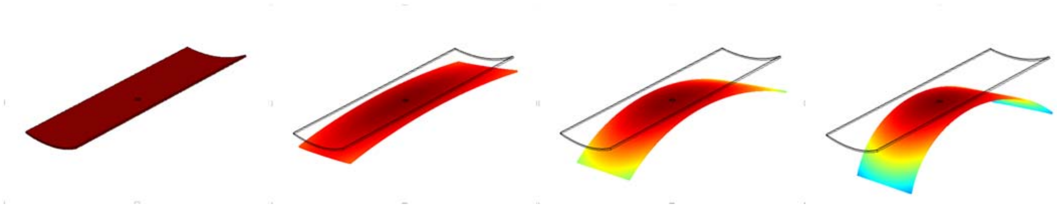

Supplementary Figure 2. Refolding behavior investigated by finite element modeling.

a

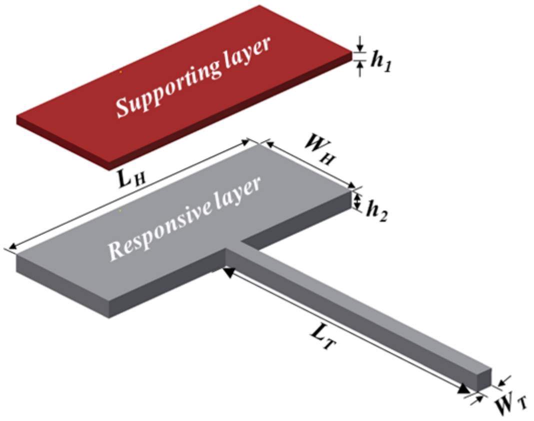

b

	Head design		Propeller mechanism
BS	Supporting layer	PS	Supporting layer
BS'	Reinforcement in supporting layer	PS'	Reinforcement in supporting layer
BR	Responsive layer	PR	Responsive layer
BR'	Reinforcement in responsive layer	PR'	Reinforcement in responsive layer

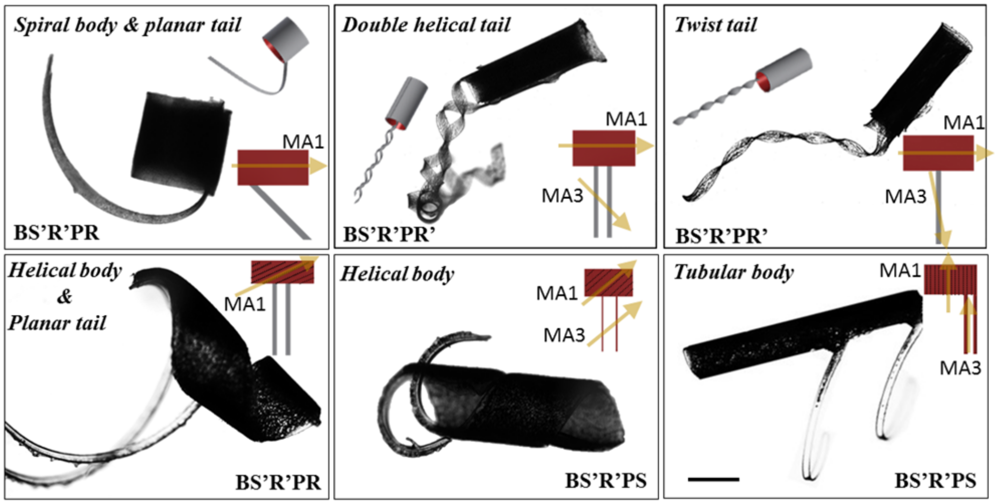

Supplementary Figure 3. Soft micromachines with complex body plans. (a) The schematic description of the geometric parameters of the 2D origami design. **(b)** The final 3D morphology of the folded layers controlled by the selective alignment of particles in different compartments durin photopatterning. The images show the versatility of the manufacturing process. The scale bar is 500µm.

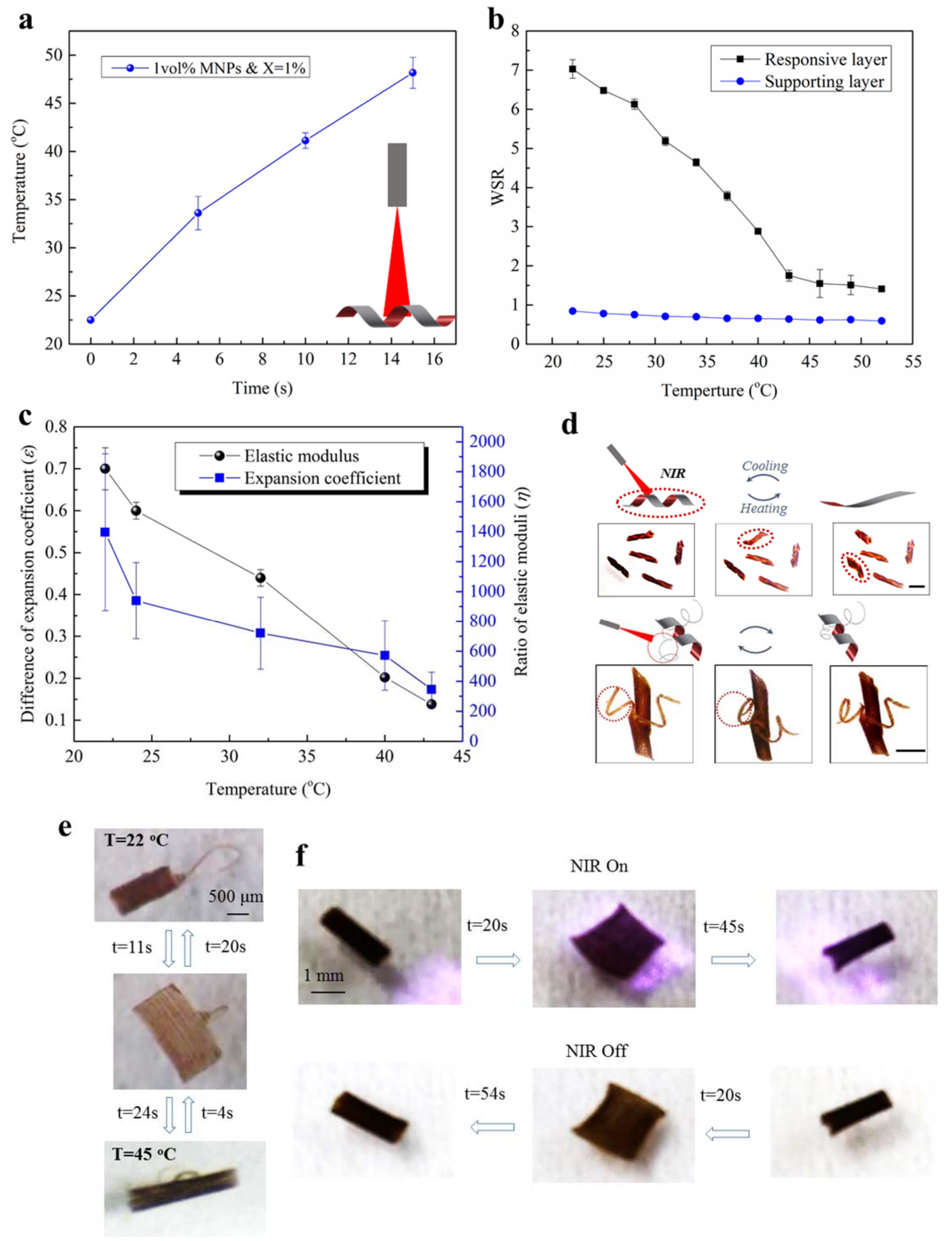

Supplementary Figure 4. Thermal response of the nanocomposites and the variations of mechanical properties with changing temperature. (a) NIR sensitivity of the nanocomposite composed of 5 wt% MNPs and weight-swelling ratio, WSR=5.5. 10 bilayer helical micromachines were heated by applying NIR. Temperature elevation was measured by a thermal image camera (Fluke

corporation, TI 400). **(b)** Temperature response of WSR of the individual responsive layer and supporting layer. **(c)** The difference in mechanical properties between the two coupling layers changing with temperature. The methods for measuring the mechanical and swelling properties are descreibed in Supplementary Notes. **(d)** Spatially control NIR for local heating. (e) The time durations of the unfolding and refolding procedures by transferring the soft micromachines between two environments with different temperatures (22˚C and 45˚C). (f) The time durations of the shape transformations of tubular bilayers triggered by exposing NIR.

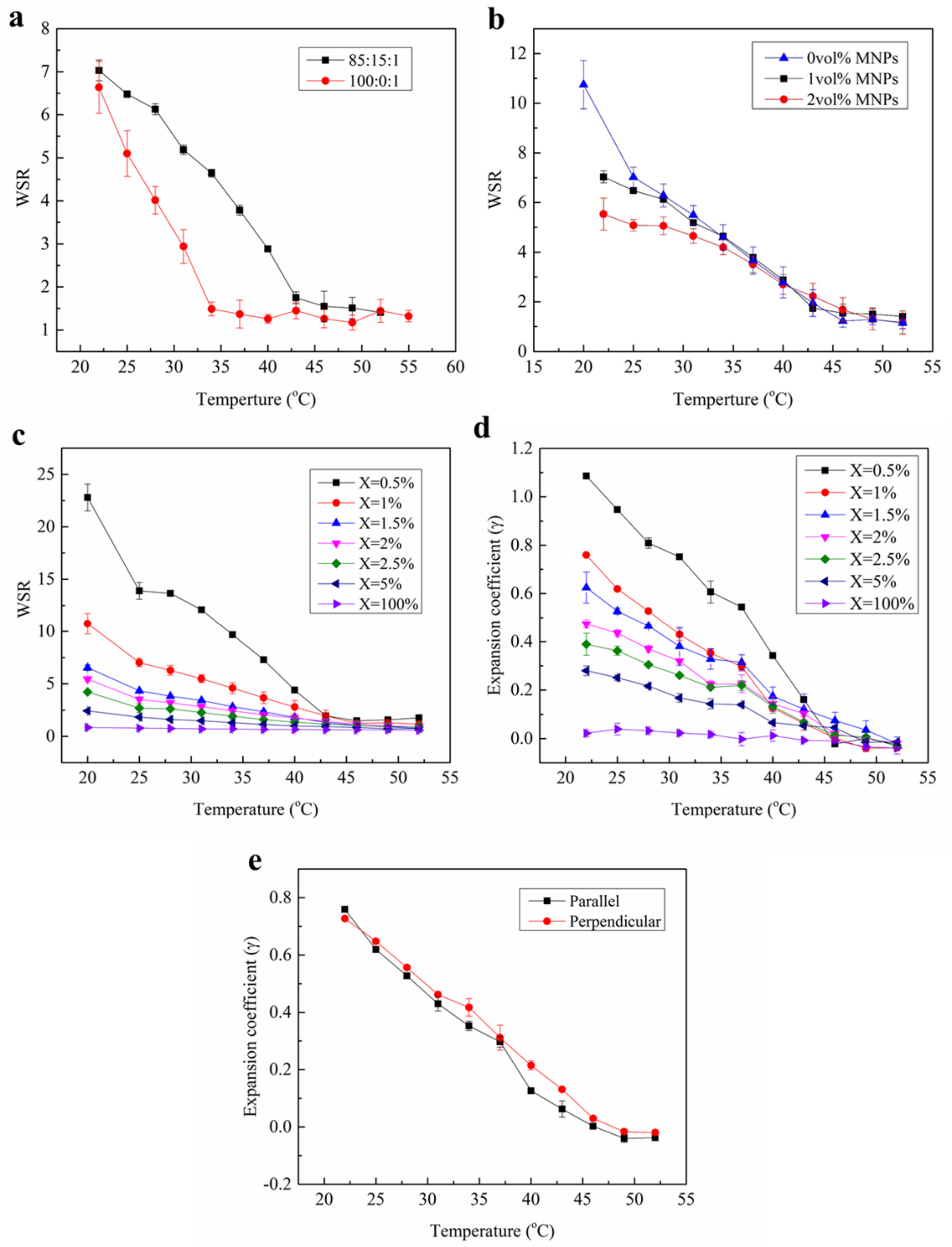

Supplementary Figure 5. **Thermal response of the hydrogel nanocomposites.** In this work the default molar ratio between NIPAAm, AAm and PEGDA is set to 85:15:1 unless otherwise stated. **(a)** Sensitivity to temperature variations of the NIPAAm hydrogel. Adding a hydrophilic co-monomer could increase the LCST of NIPPAm. **(b)** Sensitivity to temperature variations of responsive nanocomposites composed of different concentrations of MNPs. **(c)** Cross-linker ratio (X%) effect of

the sensitivity to temperature. **(d)** The expansion coefficient of the responsive gel composed of different cross-linker ratios varying with temperature. The slope of the curves is the negative thermal expansion coefficient. **(e)** The isotropic thermal expansion coefficient of the NIPAAm hydrogel.

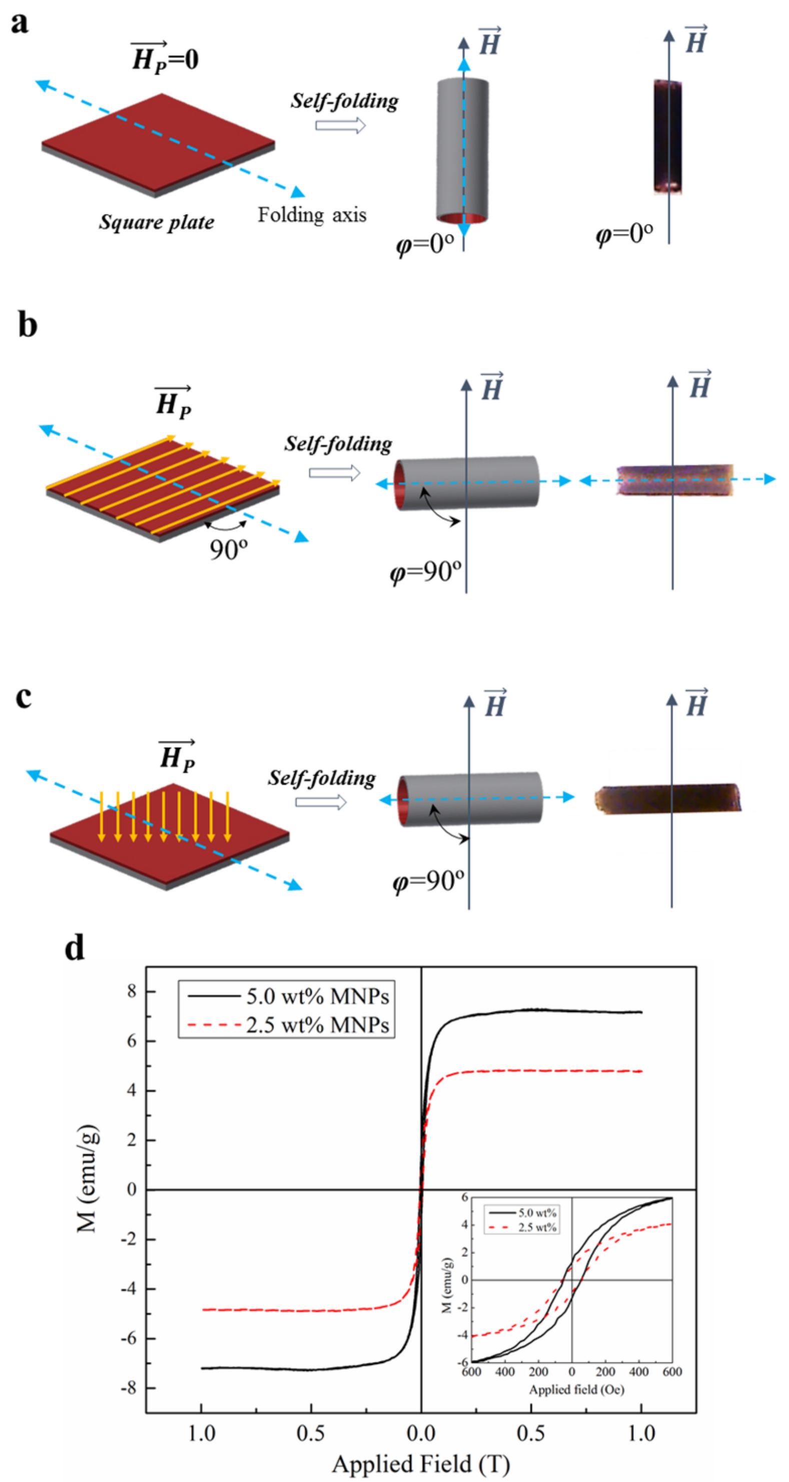

Supplementary Figure 6. Programming the magnetic anisotropy of bilayer machines in the swelling responsive layer. (a) The magnetic easy axis of bilayer folded tube without aligning the embedded MNPs is determined by its shape anisotropy, which is aligned with its long axis. $\vec{H}$ is the static magnetic field used to identify the magnetic easy axis. **(b)** The bilayer tube with planar alignment of MNPs and perpendicular to its folding axis leads to a misaligned angle φ=90°. **(c)** Out-

of-plane alignment of the embedded MNPs of bilayer sheet give rise to a radial magnetic easy-axis of the folded tube (φ=90°). **(d)** The measured magnetic properties of the nano-compsoites with different concentration of MNPs by means of vibrating sample magnetometer (VSM). The magnetic property (M) here is defined as the total magnetic moments of the embedded MNPs in the hydrogel devided by the amount of wegith of the nanocomposite.

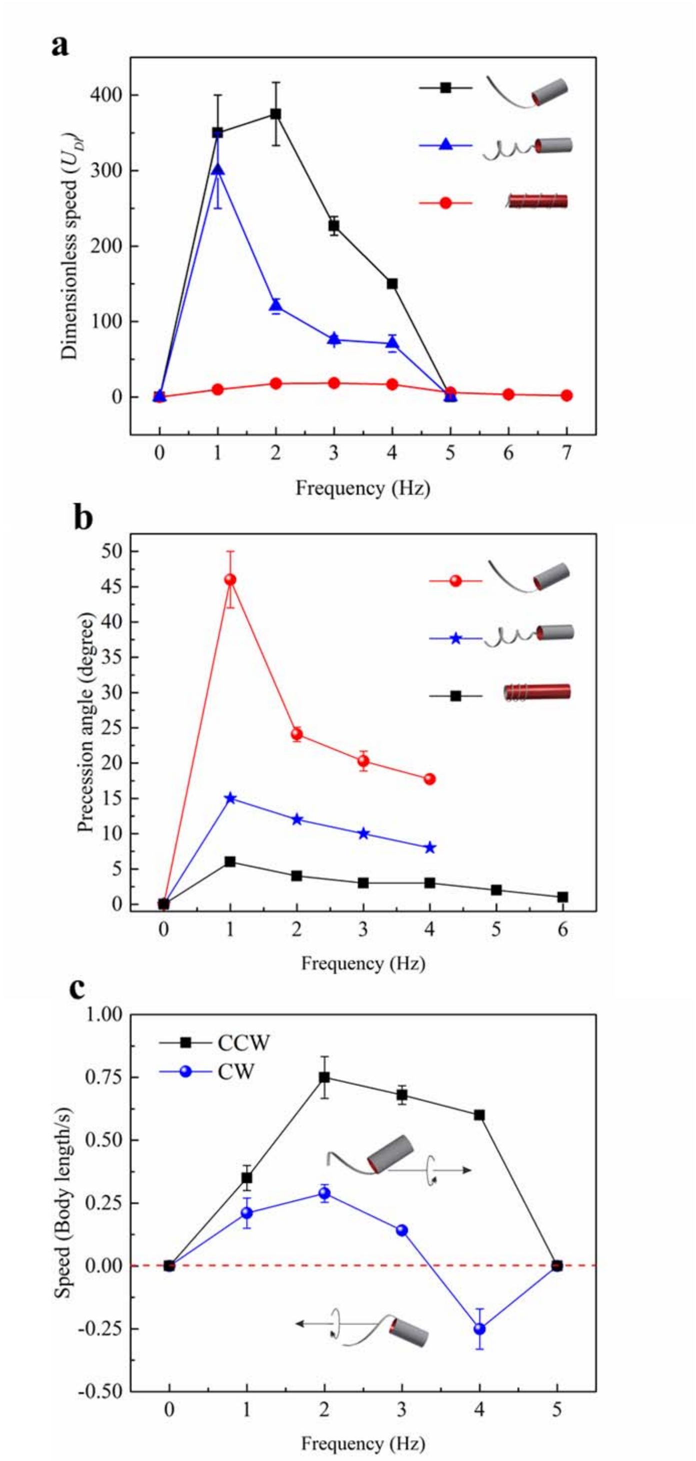

Supplementary Figure 7. Motility of the flagellated soft micromachines and speed control. (a) The dimensionless speed of different types of micromachines as a function of the frequency of rotation. **(b)** The precession angle of three different micromachines (planar tail, helical tail, tail wrapped around machine head) as a function of the frequency of rotation. **(c)** The motility of the micromachine with planar flagellum rotating in CW and CCW motion.

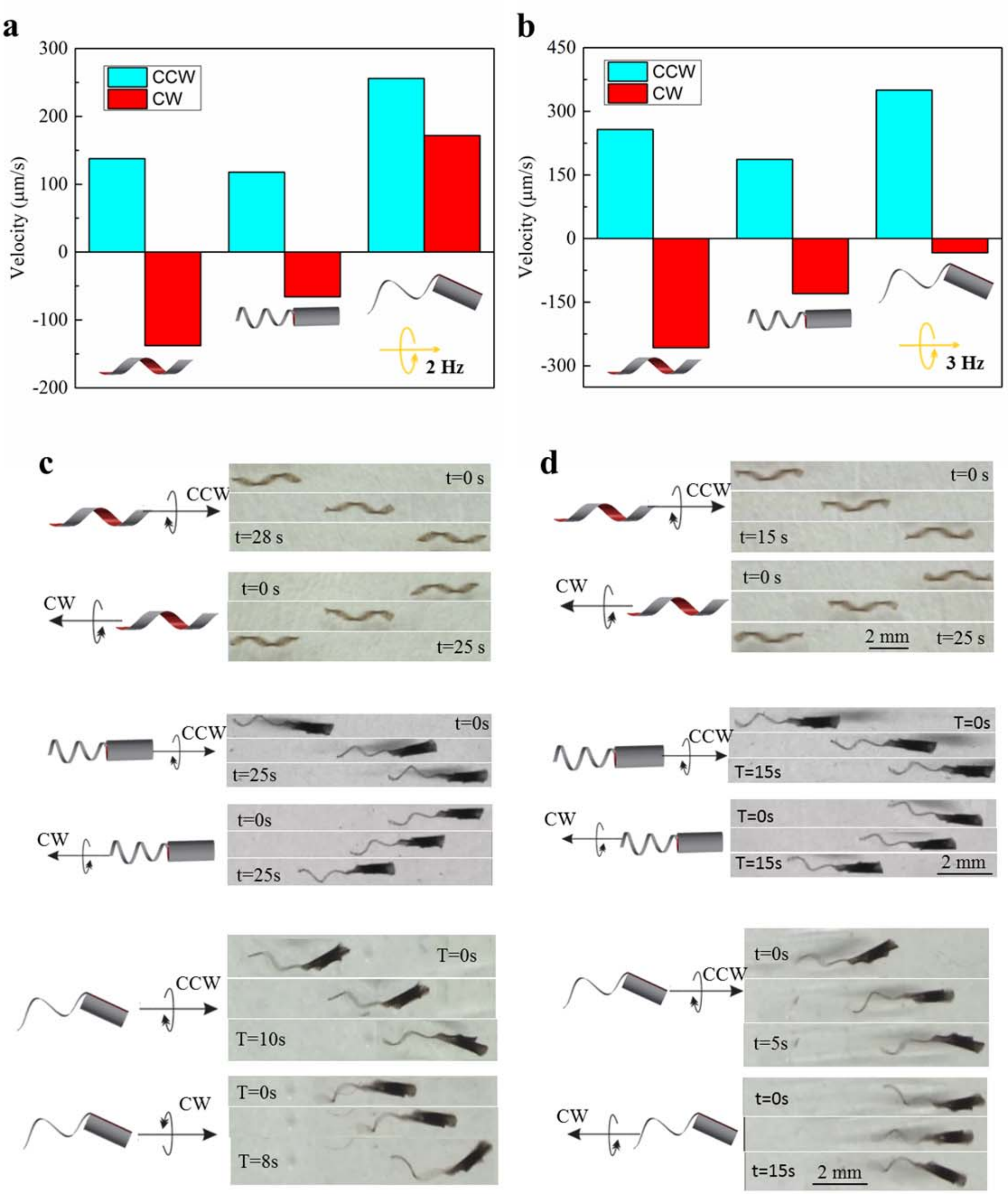

Supplementary Figure 8. The role of tail morphology on velocity control. The forward and backward velocity of three different types of micromachine (helical body, stiff helical tail, and soft helical tail) at rotating magnetic fields of **(a)** 2 Hz and **(b)** 3 Hz. The time-lapse images of the micromachines driven by rotating magnetic fields at **(c)** 2 Hz and **(d)** 3 Hz.

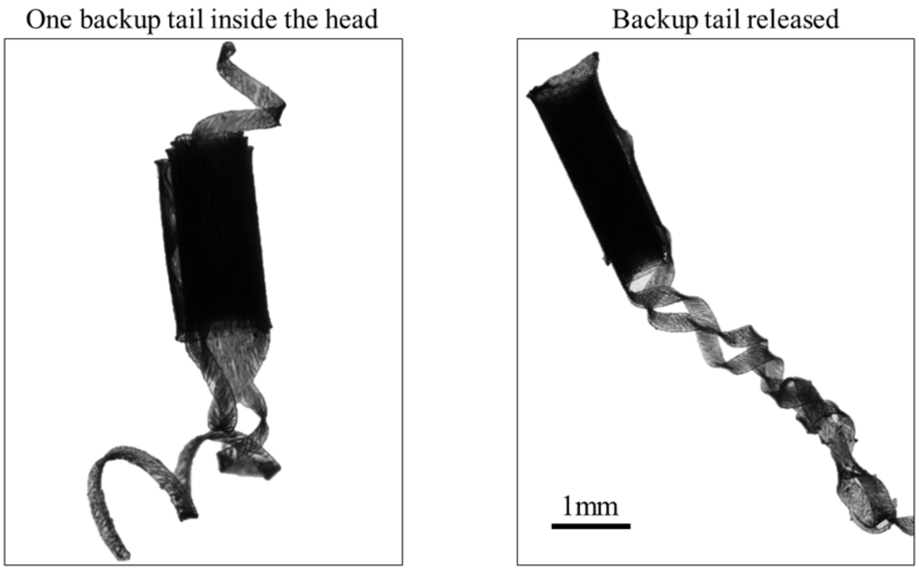

Supplementary Figure 9. Extracting hidden compartments. Substitute or extra tail hidden inside the head can be released on-demand by unfolding the tubular head with NIR exposure. The machine folds into a functional machine that has a new tail once the temperature goes back to the original level.

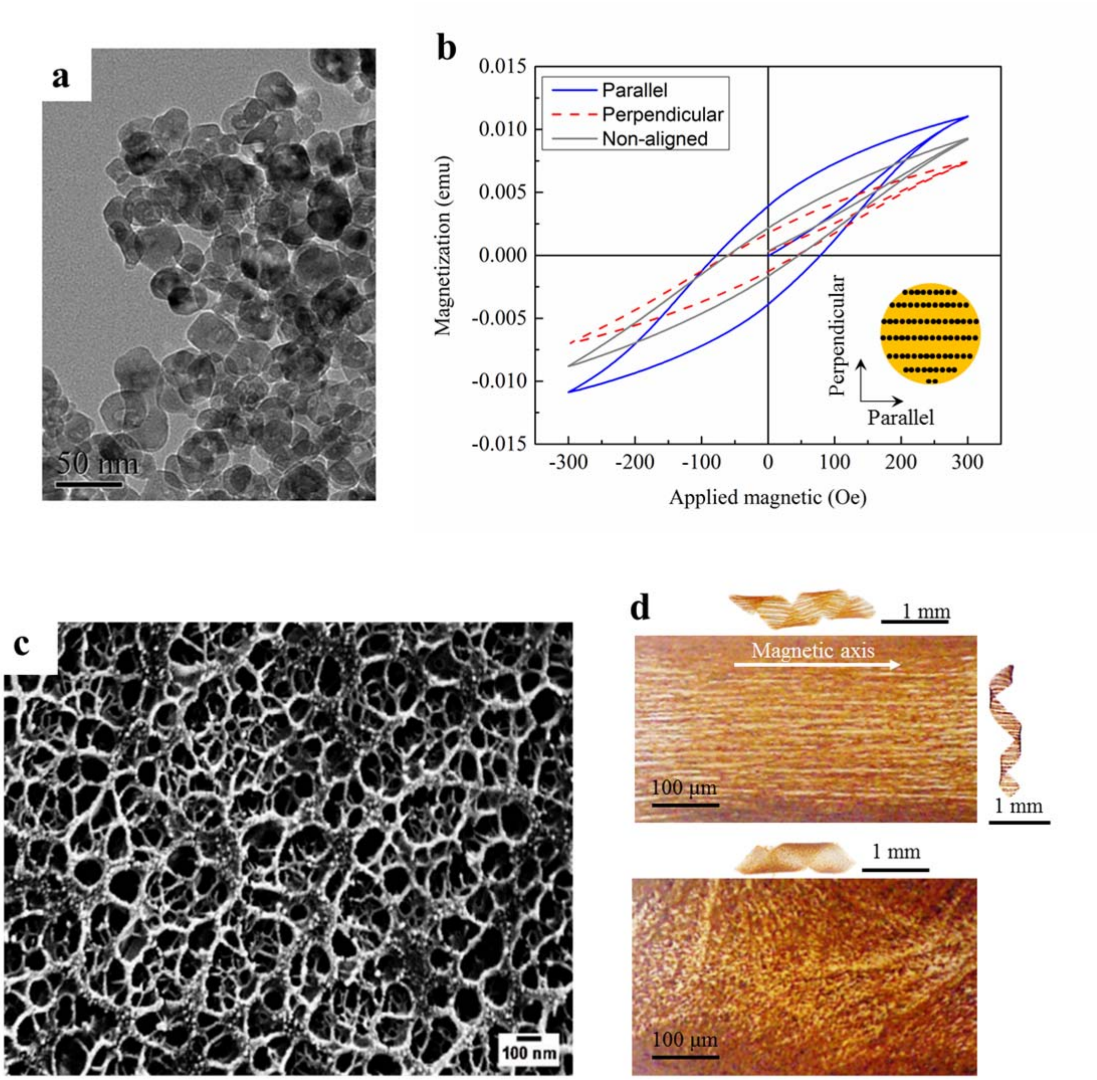

Supplementary Figure 10. The characterization of the magnetic nanocomposites. (a) Transmission electron microscope (TEM) image of the magnetic nanoparticles. The scale bar is 50 nm. (b) Magnetization of the magnetic nanocomposites, with and without particle alignment, in the parallel and perpendicular direction with respect to the magnetic axis. (c) The low temperature scanning electron microscope (Cryo-SEM) image of the network of the hydrogel nanocomposite. The white dots correspond to the magnetic nanoparticles dispersed along the fibers. (d) Optical images of the helical micromachines at different magnification with and without particle alignment.

Supplementary Table 1. Dimensions of the FSM

2D designing parameters	Defined dimensions (μm)
L_H	1500
W_H	500
L_T	750
W_T	50
h_1	10
h_2	30

Supplementary Table 2. Mechanical properties of hydrogel bilayer

Mechanical properties	WSR	Expansion coefficient	Elastic modulus (kPa)
Supporting layer	0.68±0.055	0.022±0.014	9600.84±3181.05
Responsive layer, 1vol% MNP & X=1% at 20°C	6.7±0.032	0.6±0.023	11.44±3.21

Supplementary Note 1

Finite element modeling of self-folding and refolding

We used commercial FEM software COMSOL Multiphysics® 5.0 to simulate the preferential folding of the bilayer machine body attaching with a flagellum at different locations. Due to the high swelling properties of the hydrogel, we use a neo-Hookean hyper-elastic material model to simulate the large deformation of the hydrogel bilayer. The material parameters used for the two coupling layers are shown in Supplementary Table 2. We placed the fixed anchor on the corner and middle of the long side, respectively as the location of flagellum. In the case of micromachine without flagellum, we set the fixed anchor at the central point of the rectangular bilayer sheet. The initial bending curvature indicated the preferential folding direction of the bilayer machine bodies.

The same model used in edge effects of the bilayer was also applied to simulate the refolding behavior of the bilayer. A bilayer plate was designed to be a pre-buckling rectangular plate with defined curvature as a folded bilayer, and a fixed anchor was set at the center of top layer. Because the thermal expansion coefficient of the swelling responsive layer is significantly higher than the non-swelling supporting layer, the responsive layer dominates the refolding behavior. By increasing temperature, both of the two layers experienced shrinkage. The pre-buckling plate started to bend in the opposite direction due to the shrinkage of nanocomposite layer and then went through a double-curved shape. The curvatures of the doubly curved plate are the combination of the self-folded curvature and refolded curvature, which are perpendicular to each other. As temperature increased, the self-folded curvature decreased to zero and the refolded curvature increased. Finally, the plate refolded along the opposite side and perpendicular to the self-folded axis (Supplementary Fig. 2).

Characterization of the swelling behavior

The equilibrium weight-swelling ratio (WSR) is defined as

$$\mathrm{WSR} = \frac{m_s - m_d}{m_d} \quad (1)$$

where m_s and m_d are the masses of the fully swollen and dried hydrogel, respectively. A digital micro-camera (Dnt Gmbh, Germany) was used to record the dimensional change. The expansion coefficient is defined as

$$\gamma = \frac{D_s - D_f}{D_f} \quad (2)$$

where D_f and D_s are the sizes of the hydrogel structures before and after swelling, respectively.

Mechanical Properties of Hydrogel layers

The mechanical properties of the non-swelling hydrogel (PEGDA) and the swelling responsive hydrgogel were analyzed by measuring their expansion coefficient and elastic modulus. The elastic modulus of the NIPAAm and PEGDA hydrogels were measured by uniaxial tensile test tool (242 Actuator, MTS system corp, Eden Praire, MN, USA) by using single layer sheets ($50\times10\times2.5$ mm).

Supplementary Note 2

Thermal analysis of the hydrogel nanocomposites

Temperature dependent swelling behavior of the responsive nanocomposites and the non-responsive PEGDA gel were measured by gravimetric and image methods. The gel disks (minimum three samples per type) were produced and incubated in a water bath (Julabo, Germany) at temperature of 22°C to 55°C. At predefined time intervals (minimum eight hours to ensure swelling equilibrium), the samples were removed from the water, carefully wiped residual water, and their weight and dimensions were recorded. Supplementary Fig. 5 shows the relationships of hydrogel with different material compositions between the swelling behaviors and temperature. Previous work has shown that the transition temperature can be adjusted by changing the type (hydrophobic vs hydrophilic) and concentration of co-polymers incorporated into the nanocomposite. For example, as shown in Fig. 5a, by removing the hydrophilic co-polymer (Acrylamide, AAm) from the nanocomposite solution, we

can decrease the transition temperature from 45 ˚C to 32 ˚C. A further reduction in transition temperature is achievable (LCST=9.8˚C) by incorporating a hydrophobic co-polymer (N-tertbutylacrylamide, NtBAAm) into the solution with the ratio of 50:50 between NIPAAm and NtBAAm[1,2]. Supplementary Fig. 5b, shows the thermo-responsive WSR of nanocomposites with various concentrations of MNPs measured at different temperature. Supplementary Fig. 5c, and 5d, show the WSR and the expansion coefficient of the NIPAAm hydrogel composed of various cross-linker ratios measured at different temperatures. The slopes of the Supplementary Fig. 5d, represent the thermal expansion coefficient of the thermo-responsive hydrogel. The thermal expansion coefficient of the responsive nanocomposite is constant before the temperature of the hydrogel reaches its LCST. The sensitivity to temperature of the responsive nanocomposite decreases with the cross-linker ratio of the hydrogel. Supplementary Fig. 5e, shows that the thermal expansion of the NIPAAm hydrogel are isotropic.

Supplementary Note 3

Characterization of the reinforced hydrogel nanocomposites

The magnetic nano-particles (MNPs) are commercially available, and employed without further modification. The purity of the iron oxide nanoparticles (Fe_3O_4) is up to 98%, and they are coated with 1 wt% polyvinylpyrrolidone (PVP) to prevent agglomeration in the pre-gel solution. Transmission electron microscopy (TEM) imaging shows that the average size of the nanoparticles is around 30 nm (Supplementary Fig. 10a). We performed vibrating sample magnetometer (VSM) measurements with a scanning magnetic range between -30 mT and 30 mT, which covers the magnetic fields used in all of our experiments (Supplementary Fig. 10b). The VSM results show that the largest hysteresis loop and magnetization are in the direction parallel to the alignment of MNPs and the smallest ones are in the direction perpendicular to the alignment. The hysteresis loop appears between the parallel and perpendicular alignment for the random distribution. Therefore, it is safe to claim that the alignment of MNPs determines the magnetic anisotropy of the nanocomposite. In addition, we performed low temperature scanning electron microscopy (Cryo-SEM), and the images show that

nanoparticles are uniformly distributed throughout the hydrogel polymer network (Supplementary Fig. 10c). Finally, we also provide the optical micrographs of the hydrogel nanocomposites with and without magnetic alignment. Images clearly show that the MNPs are aligned within the helical micromachine along the programmed axis, in both parallel and perpendicular directions (Supplementary Fig. 10d).

Supplementary references

1 Klouda, L. & Mikos, A. G. Thermoresponsive hydrogels in biomedical applications - a review. *European journal of pharmaceutics and biopharmaceutics: official journal of Arbeitsgemeinschaft fur Pharmazeutische Verfahrenstechnik e.V* **68**, 34-45 (2008).

2 Doorty, K. B. *et al.* Poly(N-isopropylacrylamide) co-polymer films as potential vehicles for delivery of an antimitotic agent to vascular smooth muscle cells. *Cardiovascular Pathology* **12**, 105-110 (2003).
